# The impact of pre-existing immunity on the emergence of within-host immune-escape mutations in Omicron lineages

**DOI:** 10.1099/jgv.0.002108

**Published:** 2025-05-13

**Authors:** Muna N. Ahmed, Ummay Salma Abu Habib, Abdallah M. Abdallah, Mohamed M. Emara, Arnab Pain, Asmaa A. Althani, Gheyath K. Nasrallah, Hadi M. Yassine, Hebah A. Al-Khatib

**Affiliations:** 1 Biomedical Research Center, QU Health, Qatar University, Doha, P.O. 2713, Qatar; 2 College of Health Sciences, QU Health, Qatar University, Doha, P.O. 2713, Qatar; 3 College of Medicine, QU Health, Qatar University, Doha, P.O. 2713, Qatar; 4 Pathogen Genomics Laboratory, Bioscience Program, Biological and Environmental Science and Engineering (BESE), King Abdullah University of Science and Technology (KAUST), Thuwal, 23955-6900, Saudi Arabia

**Keywords:** immune pressure, quasispecies, SARS-CoV-2, vaccination, virus evolution

## Abstract

The Omicron variant of SARS-CoV-2 circulating amongst highly immunized populations is anticipated to induce immunological pressures, potentially compromising existing immunity. This study investigates vaccine-induced immunity’s impact on within-host diversity of Omicron variants and evaluates sub-consensus mutations at spike protein antigenic sites. Next-generation sequencing assessed the within-host diversity of 728 Omicron-positive samples (421 vaccinated; 307 unvaccinated). Quantitative analysis revealed limited vaccine impact, regardless of lineage, vaccine type or doses. Non-lineage mutations (39, 33 and 25 in BA.2*, BA.4* and BA.5* lineages, respectively) were detected, some showing higher incidence in vaccinated individuals. Six mutations detected at sub-consensus levels at antigenic sites suggest increased immune pressure on the spike protein in vaccinated individuals. Four high-prevalence antigenic mutations, absent from global GISAID sequences, were identified. Although within-host diversity did not significantly differ between vaccination statuses, detected mutations suggest that vaccine-induced immunity may influence within-host mutation patterns.

## Data Availability

Raw sequencing data are deposited in the National Center for Biotechnology Information. The accession numbers of all submitted sequences are available in Table S1.

## Introduction

The emergence of SARS-CoV-2 in late 2019 has resulted in the most devastating health crisis of the twenty-first century. The World Health Organization (WHO) declared the outbreak a global health pandemic in March 2020. The pandemic has so far resulted in more than 700 million cases and 7 million deaths worldwide [1]. The advances in sequencing and vaccine technology platforms have facilitated the development of vaccines in an unprecedented timeframe. The implementation of vaccine programmes has successfully reduced the burden of the disease and saved millions of lives [2,3]. On 5 May 2023, the WHO declared the end of COVID-19 as a global health emergency. However, the continuous evolution of the virus and the emergence of new variants with reduced sensitivity to vaccines is still a major health concern. The WHO has categorized the variants based on their pathogenicity, transmissibility and immune evasion characteristics into ‘Variants of Concern’ (VOC) and ‘Variants of Interest’ [4].

The latest emerging VOC is the Omicron variant that appeared in November 2021 in South Africa [5]. The emergence of the Omicron variant was responsible for the surge in the number of COVID-19 cases in early 2022 [6,7]. Omicron variants demonstrated the highest transmission rate (3.2 times higher) compared to other VOCs [8,9]. The higher transmissibility is thought to be associated primarily with Omicron’s ability to evade the existing immunity in individuals who have been previously infected or vaccinated [10]. In addition, Omicron infections are characterized by a higher reinfection rate and decreased disease severity, indicating distinct evolution and immune system escape mechanisms [11,12]. The variant has so far evolved into more than 300 sub-variants. The continuous evolution of the Omicron variants might lead to the emergence of vaccine-resistant mutations which might consequently compromise vaccine efficiency.

Coronaviruses, like other RNA viruses, have a high mutation rate due to the impaired proofreading activity of their RNA polymerases [13]. As these viruses replicate within hosts, they generate a cloud of genetically diverse populations referred to as quasispecies. The role of within-host mutations in virus evolution has been investigated in SARS-CoV-2-infected patients [14]. Studies investigating within-host quasispecies in immunocompromised patients showed strong evidence of within-host evolution of certain mutations which have eventually become fixed in the virus genome [15,16]. Studies conducted on immunocompetent patients have also revealed variable levels of within-host diversity that change throughout the infection [14,17,19]. The within-host evolution of SARS-CoV-2 is shaped by host and virus factors such as the host’s immunological status, host age and viral load [17,20, 21]. The immune pressure is thought to be the major factor affecting virus evolution. The selection pressure resulting from existing immunity is expected to trigger the virus to evolve by acquiring immune-escaping mutations in the coronavirus spike (S) protein, the main target of the immune response [22,24]. As population-wide immunity grows, studying the effect of vaccine-induced immunity on within-host diversity and mutation selection is critical [25, 26, 27]. To this end, however, there is limited knowledge about the characteristics of within-host diversity in vaccinated individuals. Omicron lineages have displayed variable levels of neutralization against ancestral SARS-CoV-2 as well as other Omicron lineages [28]. However, it is not clear whether Omicron variants exhibit different within-host mutation patterns. In this study, we evaluated the within-host mutations in vaccinated and unvaccinated Omicron-positive cases. We have also investigated the occurrence and the prevalence of the identified within-host mutations in global virus genomes.

## Methods

### Sample selection and study groups

Positive nasopharyngeal swabs were collected during the Omicron wave (May 2022 to September 2022). A total of 1,400 samples were received from the virology laboratory at Hamad Medical Corporation and stored in aliquots in the Biomedical Research Center at Qatar University. Upon receival, samples were aliquoted and stored at −80 °C. Demographical and clinical data including age, sex, sample collection date, Ct values, disease severity and vaccination status (vaccine type and number of doses) were also obtained. Only samples that belong to patients aged 18–55 with no history of infection were included in the study. Samples from severely ill and asymptomatic individuals were excluded. Samples with Ct values between 15 and 25 were selected for sequencing (*n*=728 samples). All patients were infected within a year of their last vaccination. Based on sequencing results, samples were grouped based on Omicron sub-lineage, vaccination status (vaccinated vs unvaccinated), vaccine type (mRNA-1273 and BNT162b2) and the number of vaccine doses.

#### Next-generation sequencing

Viral RNA was extracted from 200 µl of viral transport medium using the MGIEasy Nucleic Acid Extraction Kit on the MGISP-960 High-throughput Automated Sample Preparation System (MGI, China). Sequencing of the SARS-CoV-2 genome was performed as per the COVID-19 Midnight protocol (SQK-RBK110.96 and EXP-MRT001) available from Oxford Nanopore Technology (ONT, UK). In brief, RNA samples were diluted based on Ct values: a 10× dilution was done for samples that have Ct values between 15 and 18, whilst no dilution was done for samples having Ct values between 18 and 25. A total of 94 samples were pooled in each flow cell in addition to a negative (no RNA) and a positive RNA control. The reverse transcription step was performed using Luna Script RT SuperMix (NEB, UK). The produced cDNA was PCR-amplified using two sets of primers (Midnight-ONT/V3) to ensure full coverage of the virus genome. Each sample library was then barcoded using a unique oligonucleotide sequence. Barcoded libraries were pooled together and purified using SPRI beads (Beckman Coulter, USA). The pooled library was quantified using the Qubit dsDNA HS assay kit (Invitrogen, USA). A total of 400 ng of library was loaded on an R9.4.1 flow cell and run on the GridION platform for 24 h or until 2.0–2.5 GB of data was obtained. All sequence data have been deposited in the National Center for Biotechnology Information (PRJNA1212817). Detailed protocols for the sequencing steps are available on Nanopore Community (https://nanoporetech.com/community).

### Bioinformatics analysis

The generated FASTQ files were analysed using the Nextflow workflow. Following demultiplexing, the sequencing reads were filtered based on read length (~1,200 bp) and read quality (>7 Q-score). Filtered reads were aligned against the reference genome (MN908947.3) to reconstruct the consensus sequence and determine the Omicron lineage. Samples were then sub-divided based on the Omicron sub-lineage into 14 groups. Only sequences that displayed coverage of >90% (*n*=409) were subjected to deep sequence analysis to assess the within-host diversity and mutations. Of the samples carried forward for deep sequence analysis, 110 were from unvaccinated individuals and 299 from vaccinated individuals (Table 1). For within-host mutation analysis, alignment files (BAM files) were re-analysed using the LoFreq variant analysis tool [29] to call mutations with minor allele frequencies (MAFs) higher than 5% (MAF >0.05). The median sequencing depth of samples ranged from 200 to 12,130 reads per sample. Mutations were further filtered based on depth (>100×). False-positive mutations were filtered out if detected in the positive RNA control (known sequence). Additional filtration of false-positive mutations was performed by excluding mutations that had significant strand bias values of less than 0.05 (*P*-value<0.05) [17].

**Table 1.** Number of unvaccinated and vaccinated individuals from each Omicron sub-lineage

Lineages	Unvaccinated individuals	Vaccinated individuals
**BA.2**	11	25
BA.2.40.1	2	4
BA.2.56	3	0
BA.2.75.1	2	0
BA.2.75.2	3	11
BA.2.76	0	4
**BA.4**	0	12
BA.4.1	8	14
**BA.5**	9	16
BA.5.1	4	18
BA.5.2	45	135
BA.5.2.1	16	41
BA.5.3	2	4
BA.5.3.1	5	15
**Total**	110	299

### Assessment of global prevalence of mutations

The global prevalence of mutations identified in this study was investigated by comparing them to sequences with a sequence length of more than 20,000 bp that were deposited worldwide in GISAID during the period of May 2022 to September 2022.

We compared our data to 237,857 sequences for BA.2, 2,297 sequences for BA.2.40.1, 2,558 sequences for BA.2.75.2, 41,235 sequences for BA.4, 65,843 sequences for BA.4.1, 19,078 sequences for BA.5, 209,594 sequences for BA.5.1, 223,883 sequences for BA.5.2, 242,592 sequences for BA.5.2.1, 3,634 sequences for BA.5.3 and 4,963 sequences for BA.5.3.1. The data were accessed through the online interactive platform CoV-Spectrum [30].

### Statistical analysis

GraphPad Prism 9 was used to perform statistical analysis. The Shapiro–Wilk test was performed to assess normality, and Mann–Whitney and Kruskal–Wallis tests were used to evaluate the significance in within-host diversity values between the different groups. Significance was considered for *P*-values<0.05.

## Results

### Identifying circulating Omicron lineages (May 2022 to September 2022)

**Fig. 1.**
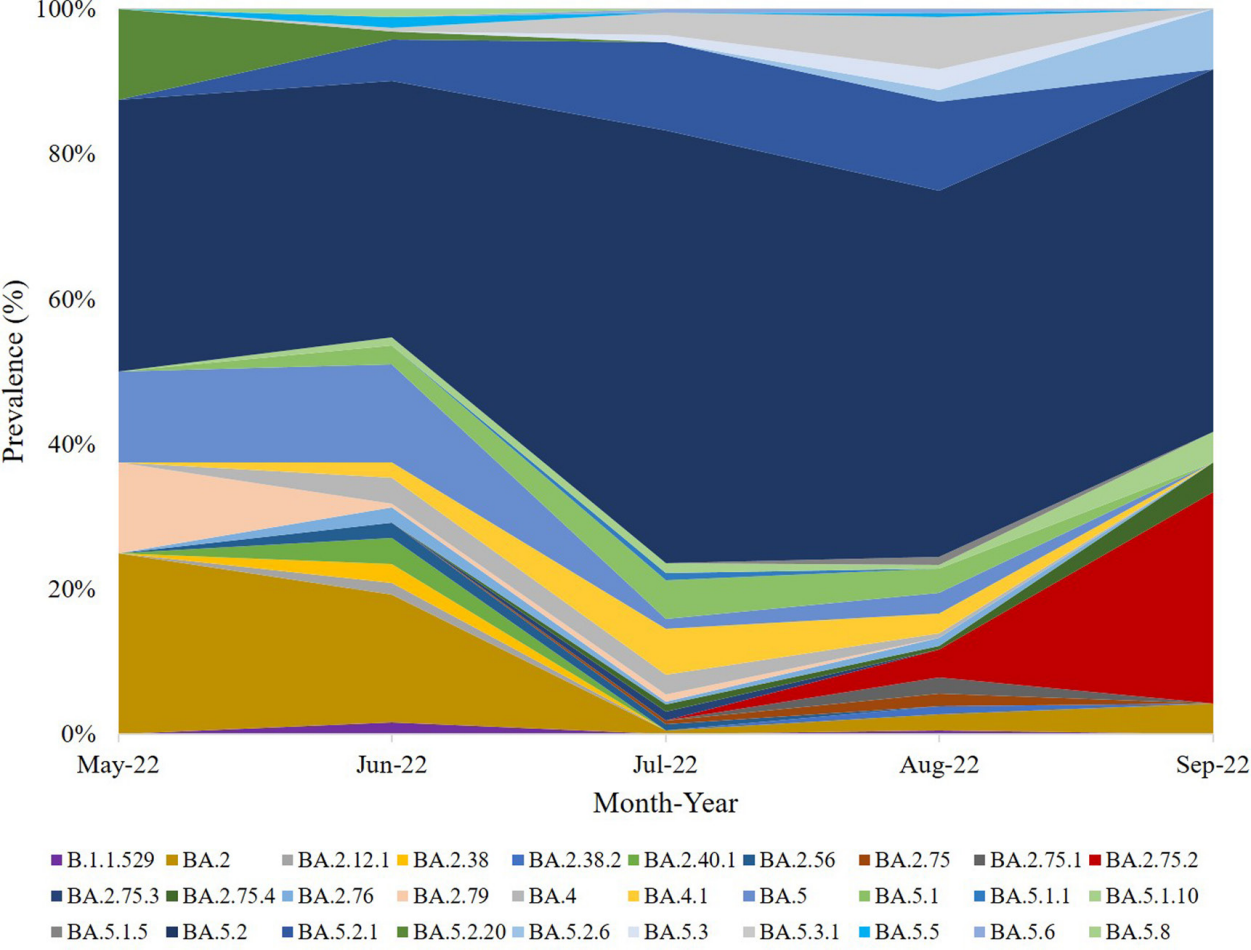
Overall prevalence of Omicron lineages circulated in Qatar during May and September 2022.

As part of the national surveillance efforts, a total of 728 samples were sequenced in Qatar during May and September 2022. Of these, 409 samples had >90 % sequence coverage of the viral genome and were considered in this study. Overall, more than 50 Omicron lineages were identified during this period. The Omicron lineage, BA.5, was the dominant Omicron lineage with 58% overall prevalence, with BA.5.2 being the most prevalent sub-lineage (44%) (Fig. 1). Few lineages were solely observed in vaccinated individuals, including three BA.2 sub-lineages (BA.2.12.1, BA.2.75.3 and BA.2.75.4) and five BA.5 sub-lineages (BA.5.1.1, BA.5.1.10, BA.5.1.5, BA.5.2.6, BA.5.5 and BA.5.6). Those lineages were particularly found in the second- and third-dose-vaccinated individuals (Fig. S1, available in the online Supplementary Material). However, these sub-lineages were excluded from further analyses due to the relatively low number of samples within these groups.

### Evaluating within-host virus diversity in Omicron samples

**Fig. 2.**
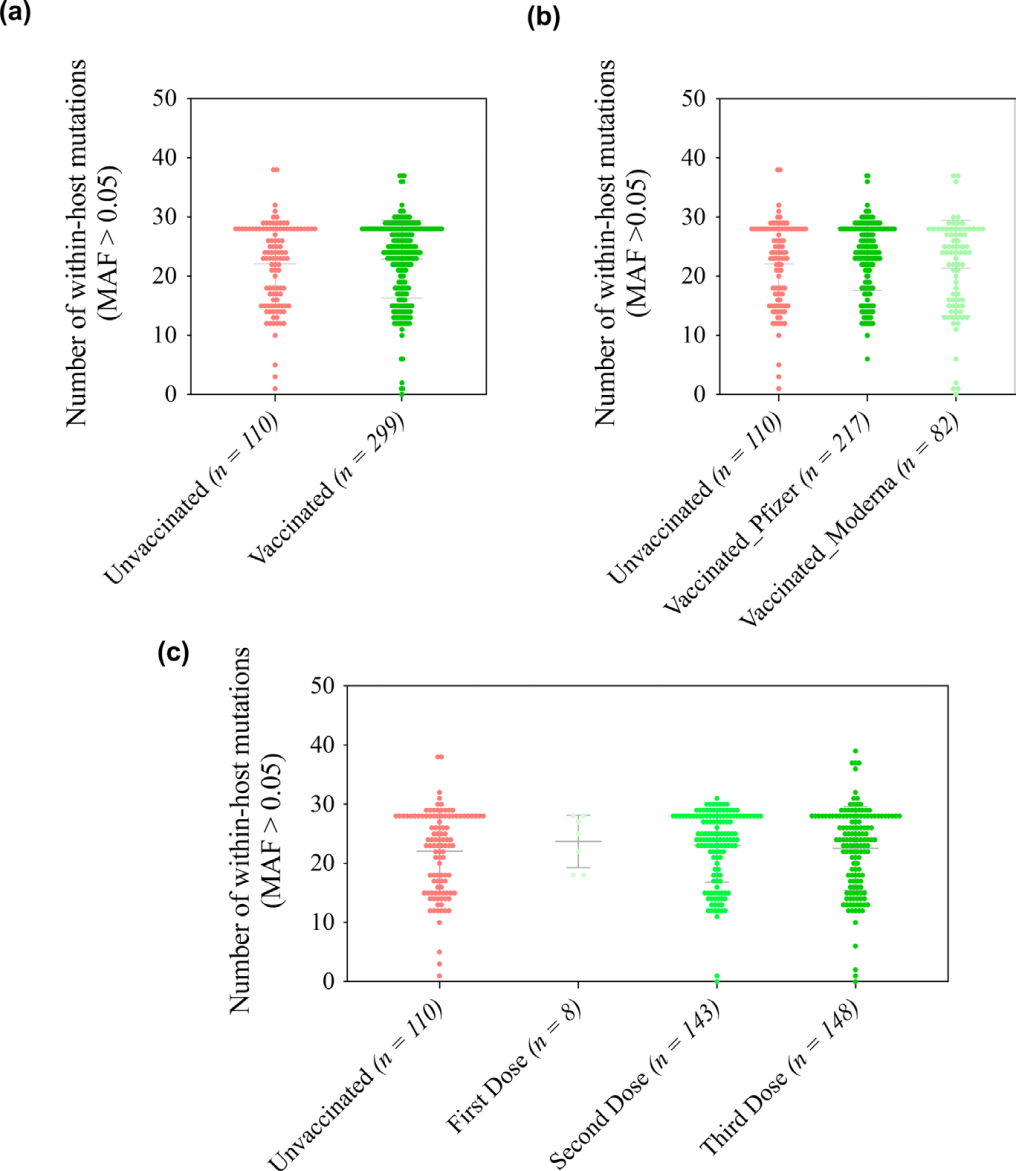
Evaluation of within-host mutations located in the S gene in Omicron-positive samples. Panels demonstrate the number of within-host mutations in (**a**) unvaccinated and vaccinated individuals, (**b**) unvaccinated and BNT162b2-vaccinated and mRNA-1273-vaccinated individuals and (**c**) unvaccinated individuals and vaccinated individuals who received one dose, two doses, three doses and four doses of vaccination. The X-axis represents the vaccination status (**a**), vaccine type (**b**) and vaccine doses (**c**). The Y-axis represents the number of within-host mutations in the whole genome with the MAF >0.05.

Analysis of within-host diversity in Omicron-positive samples was performed by assessing the number of mutations with an MAF >0.05. First, we investigated the impact of the vaccine on within-host diversity regardless of Omicron lineages. Analysis revealed no significant difference between vaccinated and unvaccinated individuals (Fig. 2a). Similarly, no significant differences were observed between mRNA-1273- and BNT162b2-vaccinated individuals (Fig. 2b). The number of vaccine doses also showed no impact on virus diversity (Fig. 2c).

**Fig. 3.**
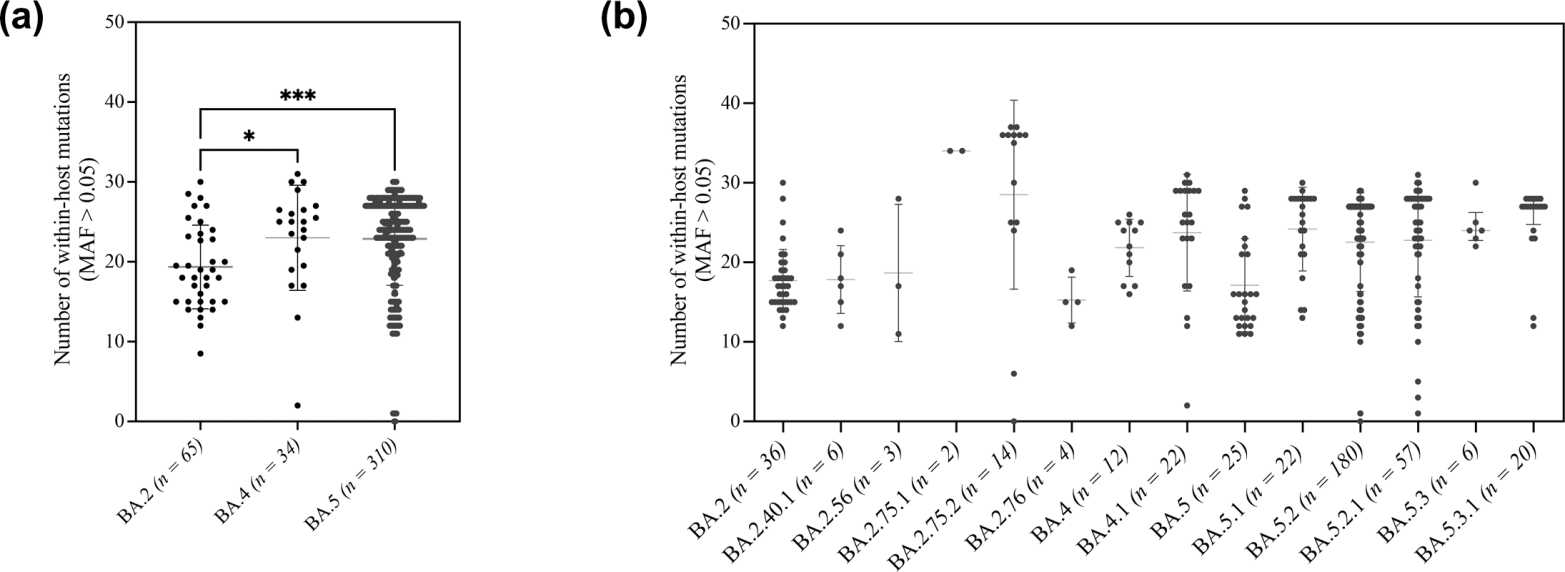
Comparison of within-host mutations in the S gene amongst different Omicron lineages. Panels demonstrate the number of within-host mutations in individuals infected with different Omicron lineages (**a**) and sub-lineages (**b**). The Y-axis represents the number of non-lineage-specific within-host mutations with MAF >0.05. Significance is indicated as * for *P*-values <0.05 and ** for *P*-values <0.001.

We then investigated the impact of vaccination on within-host diversity amongst the three most prevalent Omicron lineages and their 11 relevant sub-lineages (Fig. 3). Analysis revealed a significantly higher number of within-host mutations in individuals infected with BA.4 and BA.5 lineages compared to BA.2 (*P*-value <0.01) (Fig. 3a). Further analysis of Omicron sub-lineages showed that the highest number of within-host mutations was found amongst BA.2.75.2 samples with a median of 36.5 (26–37.75), followed by BA.5.3.1 samples [median=28 (25–29)] (Fig. 3b). A comparison of the within-host diversity amongst different Omicron lineages based on vaccination status revealed a higher number of mutations in unvaccinated individuals infected with BA.2.75.2 [median=36.5 (26–37.75)] and BA.5.2 [median=25 (15–28)] compared to vaccinated individuals.

**Fig. 4.**
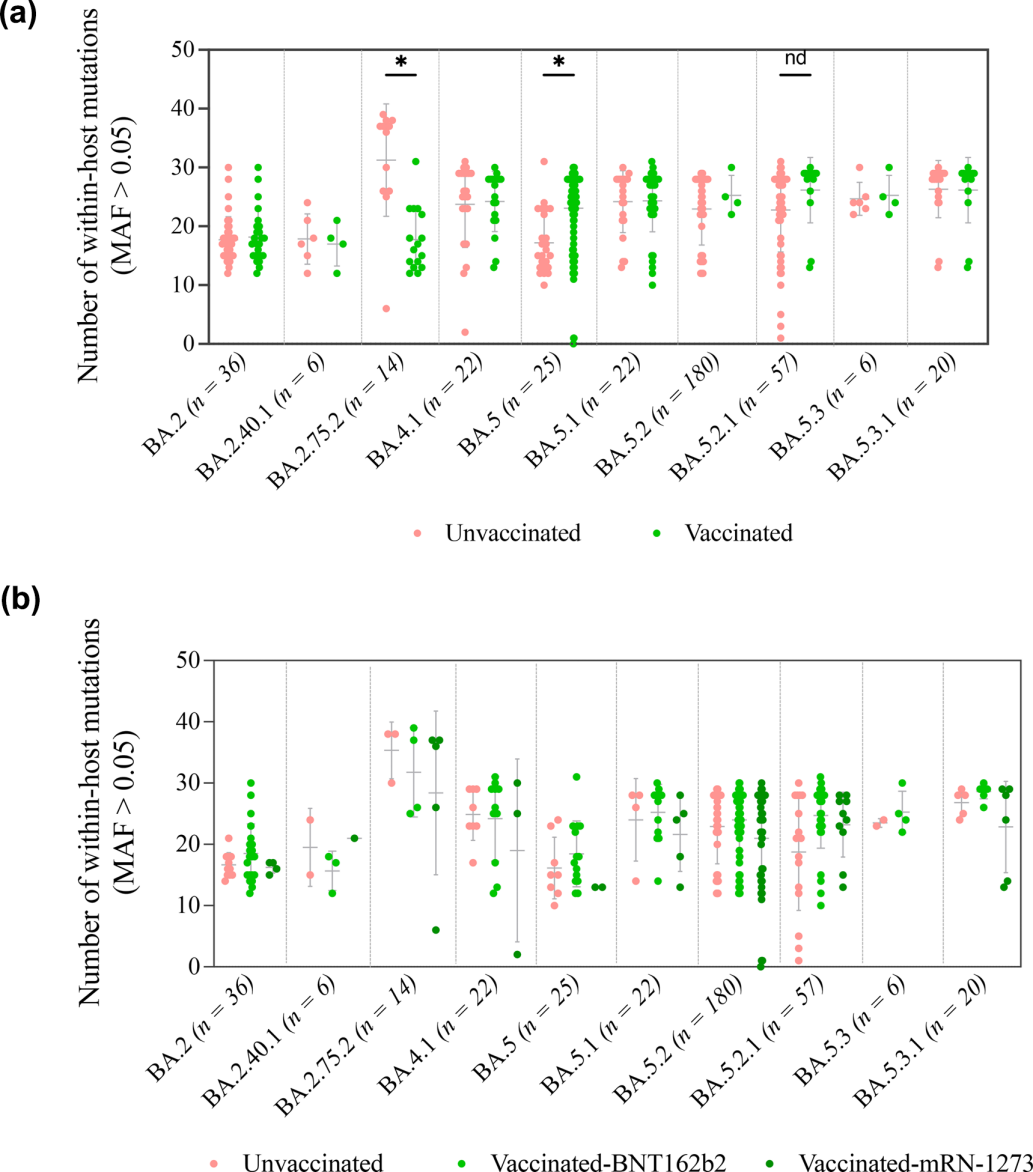
Comparison of within-host mutations in the S gene amongst different Omicron lineages. Panels describe the number of within-host mutations on the basis of vaccination status (**a**) and vaccine type (**b**). The Y-axis represents the number of non-lineage-specific within-host mutations with MAF >0.05. Significance is indicated as * for *P*-values <0.05 and ** for *P*-values <0.01.

In contrast, vaccinated individuals infected with BA.5 [median=15.5 (22.75–13)], BA.5.2 [median=25 (15–28)] and BA.5.2.1 [median=25 (21–28)] had a higher number of within-host mutations compared to unvaccinated individuals (Fig. 4a). Further analysis of samples based on vaccine type revealed a slight increase in the number of mutations in individuals vaccinated by BNT162b2 in comparison to mRNA-1273 across all lineages (Fig. 4b). Analysis based on vaccine doses did not show a significant difference in the number of within-host mutations across groups. Overall, no significant differences were observed in the within-host diversity of SARS-CoV-2 regardless of Omicron lineages, vaccine type and number of administered doses. The vaccination status of individuals showed contrasting effects on individuals infected by BA.5 and BA.5.2.1 and those infected by the BA.2.75 sub-lineage.

### Evaluating the prevalence of within-host mutations in the S protein

Here, we assessed the prevalence of the S mutations. We included non-synonymous S mutations with a minimum depth of 100× and a minor allele frequency (MAF) of 0.05 which are identified as lineage-specific mutations. We compared mutation prevalence between vaccinated and unvaccinated individuals infected with the same Omicron lineage. We analysed the prevalence of non-lineage mutations across all sub-lineages of BA.2*, BA.4* and BA.5* (Fig. S2). A total of 30 mutations were observed across all sequences of the BA.2* family. Five mutations were found in the antigenic sites within the receptor-binding domain (RBD), namely, V4451, G446S, N460K, F486S and H519P (Fig. S2A). Interestingly, F486S had a relatively higher prevalence (29%) amongst individuals vaccinated with three doses, suggesting a possible role of vaccines in the occurrence of the mutation. In sequences belonging to BA.4* sub-lineages, a total of 33 non-lineage mutations were detected, with three of them located at antigenic sites, however, with low prevalence (<20%) (Fig. S2B). Assessment of mutation prevalence across the BA.5* sub-lineages revealed a total of 25 non-lineage mutations present across different vaccination statuses and doses with a prevalence of less than 20%. Three of these mutations, K444N, H519P and A522P, were located in the S antigenic sites (Fig. S2C).

**Fig. 5.**
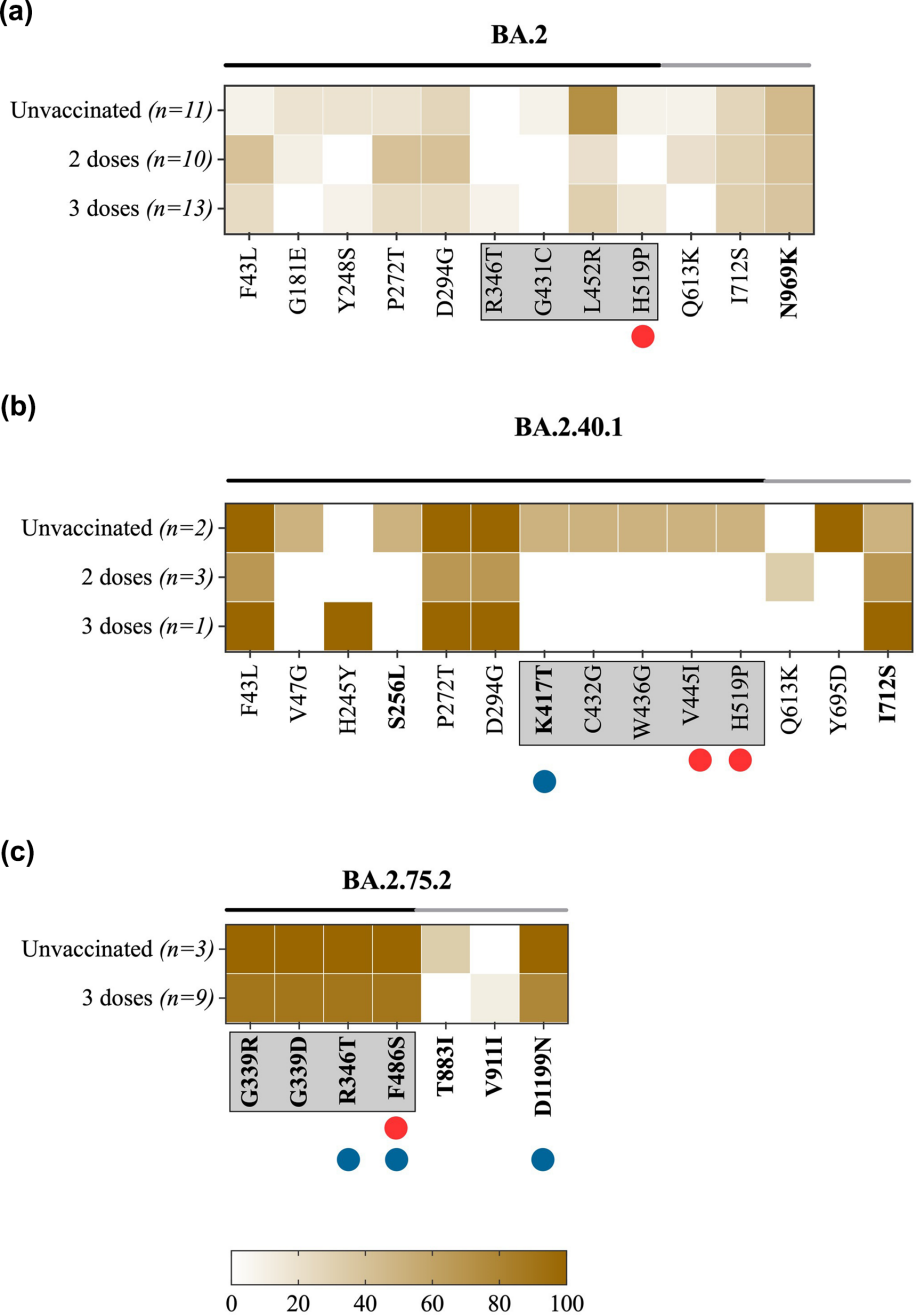
Heat map*s* demonstrating the prevalence of within-host mutations located in the S gene in (**a**) BA.2 sequences, (**b**) BA.2.40.1 sequences and (**c**) BA.2.75.2 sequences. The heat maps compare the prevalence of each mutation between unvaccinated and vaccinated individuals. Vaccinated groups were further subdivided based on the number of vaccine doses that they received into one-dose-vaccinated, two-dose-vaccinated and three-dose-vaccinated individuals. The X-axis represents the amino acid mutations in the S gene. The amino acid changes that lie in the S1 and S2 subunits are marked by black and grey lines on the top of the heat map, respectively. Mutations located in the RBD are boxed. Mutations identified at consensus levels are labelled in bold. The red circle denotes amino acid mutations in antigenic sites. The blue circle denotes mutations detected at global prevalence.

We further evaluated the prevalence of non-lineage mutations within each Omicron sub-lineage. A total of 11 sub-consensus amino acid mutations were identified in BA.2 sub-lineage sequences, one of which, H519P, was found in an antigenic site with less than 20% prevalence in unvaccinated and three-dose-vaccinated individuals (Fig. 5a). Upon investigation of this mutation at the global level, no sign of this mutation was found in GISAID data (*n*=237,857 sequences). Mutation L452R was detected with the highest prevalence amongst our BA.2 sequences (unvaccinated=73%, two-dose vaccinated=20%, three-dose vaccinated=31%). It is intriguing that global reports show this mutation to be fixed in BA.4 and BA.5 lineages and present in only 0.5% of BA.2. The BA.2 sub-lineage, BA.2.40.1, carried 14 non-lineage mutations in the S gene, three at consensus levels (Fig. 5b). Two of these mutations, V445I and H519P, were found in the antigenic sites in all the unvaccinated samples of the BA.2.40.1 sub-lineage. Another mutation, K417T, was found in the RBD of an unvaccinated individual. This mutation has been reported in 94.5% of global BA.2.40.1 sequences (*n*=2,375 sequences). The low number of BA.2.40.1 sequences (*n*=5 samples) included in the analysis must be taken into consideration. Another BA.2 sub-lineage, BA.2.75.2, possessed seven non-lineage mutations at a consensus level, five of which were detected in more than 75% of sequences of unvaccinated and two-dose vaccinated groups (Fig. 5c). One of these highly prevalent mutations, F486S, is located at an antigenic site within the receptor-binding motif and was found in more than 85% of unvaccinated and three-dose-vaccinated individuals. This mutation was found in 94.6% of GISAID sequences that belong to the BA.2.75.2 lineage (*n*=2,558 sequences). However, it is not considered a mutation of interest or concern. The RBD mutation, R346T, was also found in 92.3% of BA.2.75.2 samples. The global prevalence of this mutation in BA.2.75.2 sequences is 95.4%. Mutations G339R and G339D were detected at consensus levels at a high prevalence of >85 % but had no global records (Fig. 5c).

**Fig. 6.**
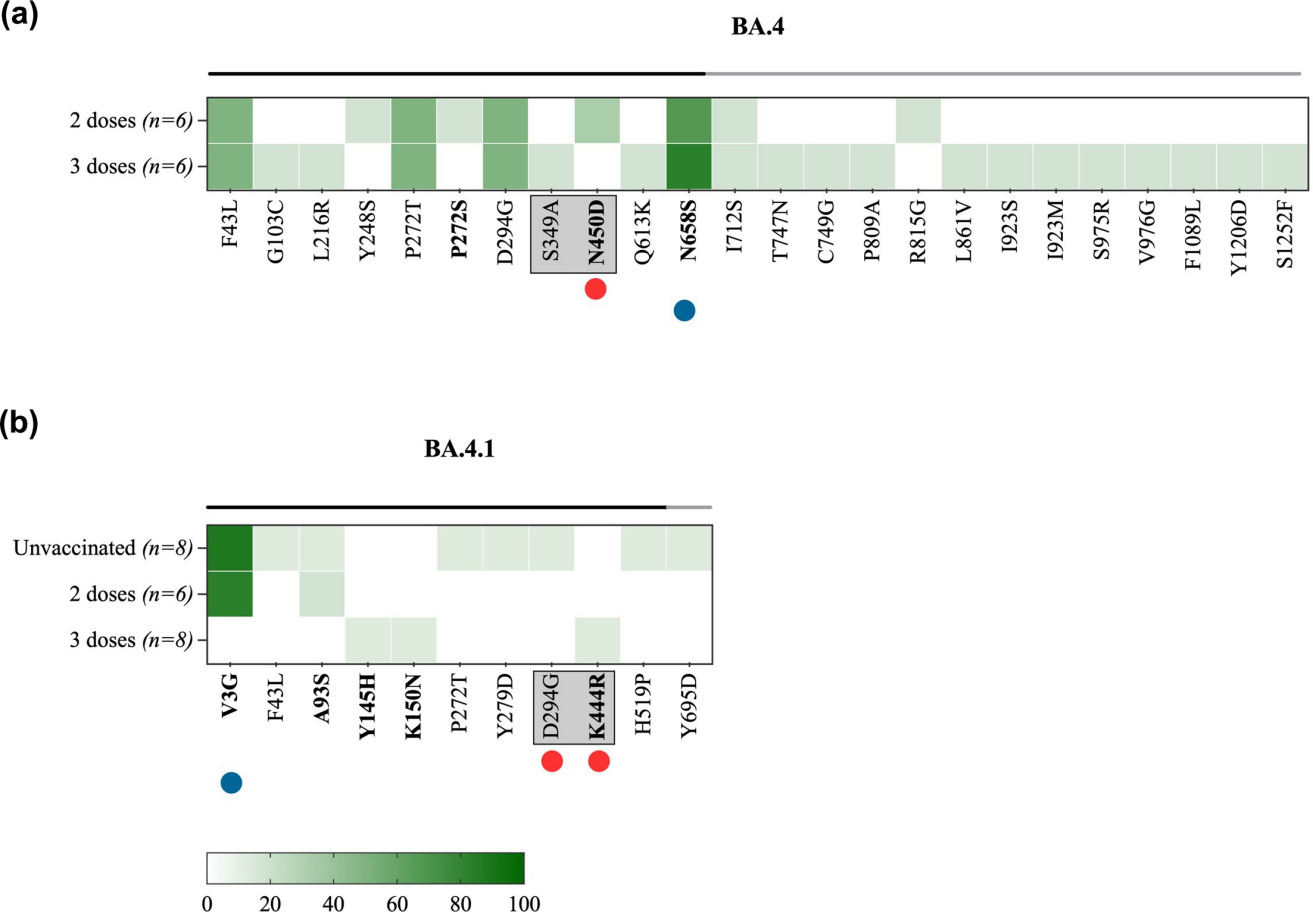
Heat maps demonstrating the prevalence of within-host mutations located in the S gene in (**a**) BA.4 sequences and (**b**) BA.4.1 sequences. The heat maps compare the prevalence of each mutation between unvaccinated and vaccinated individuals. Vaccinated groups were further subdivided based on the number of vaccine doses that they received into one-dose-vaccinated, two-dose-vaccinated and three-dose-vaccinated individuals. The X-axis represents the amino acid mutations in the S gene. The amino acid changes that lie in the S1 and S2 subunits are marked by black and grey lines on the top of the heat map, respectively. Mutations located in the RBD are boxed. Mutations identified at consensus levels are labelled in bold. The red circle denotes amino acid mutations in antigenic sites. The blue circle denotes mutations detected at global prevalence.

Analysis of BA.4 sequences revealed the presence of 22 non-lineage mutations at the sub-consensus level, exclusively in vaccinated individuals (Fig. 6a). The majority of detected mutations were found at higher prevalence amongst three-dose-vaccinated individuals in comparison to two-dose-vaccinated individuals. The only exceptions were the antigenic mutations, N450D and R815G, which were found at 33% and 17% prevalence, respectively, in unvaccinated individuals only. The most prevalent mutation in BA.4-infected individuals is N658S, found at a consensus level with more than 65% prevalence amongst both two-dose-vaccinated and three-dose-vaccinated groups (Fig. 6a). This is in line with its global prevalence of 59.8%. In the BA.4.1-positive individuals, 11 non-lineage mutations were detected. Two mutations, K444R and H519P, which are located in antigenic sites, were found in three-dose-vaccinated and unvaccinated individuals, respectively, at a prevalence of less than 15% (Fig. 6b). Mutation V3G was found at consensus levels with a prevalence of more than 80% in unvaccinated and two-dose-vaccinated individuals. The global prevalence of this mutation was also high (97.9%) amongst sequences that belong to this sub-lineage.

**Fig. 7.**
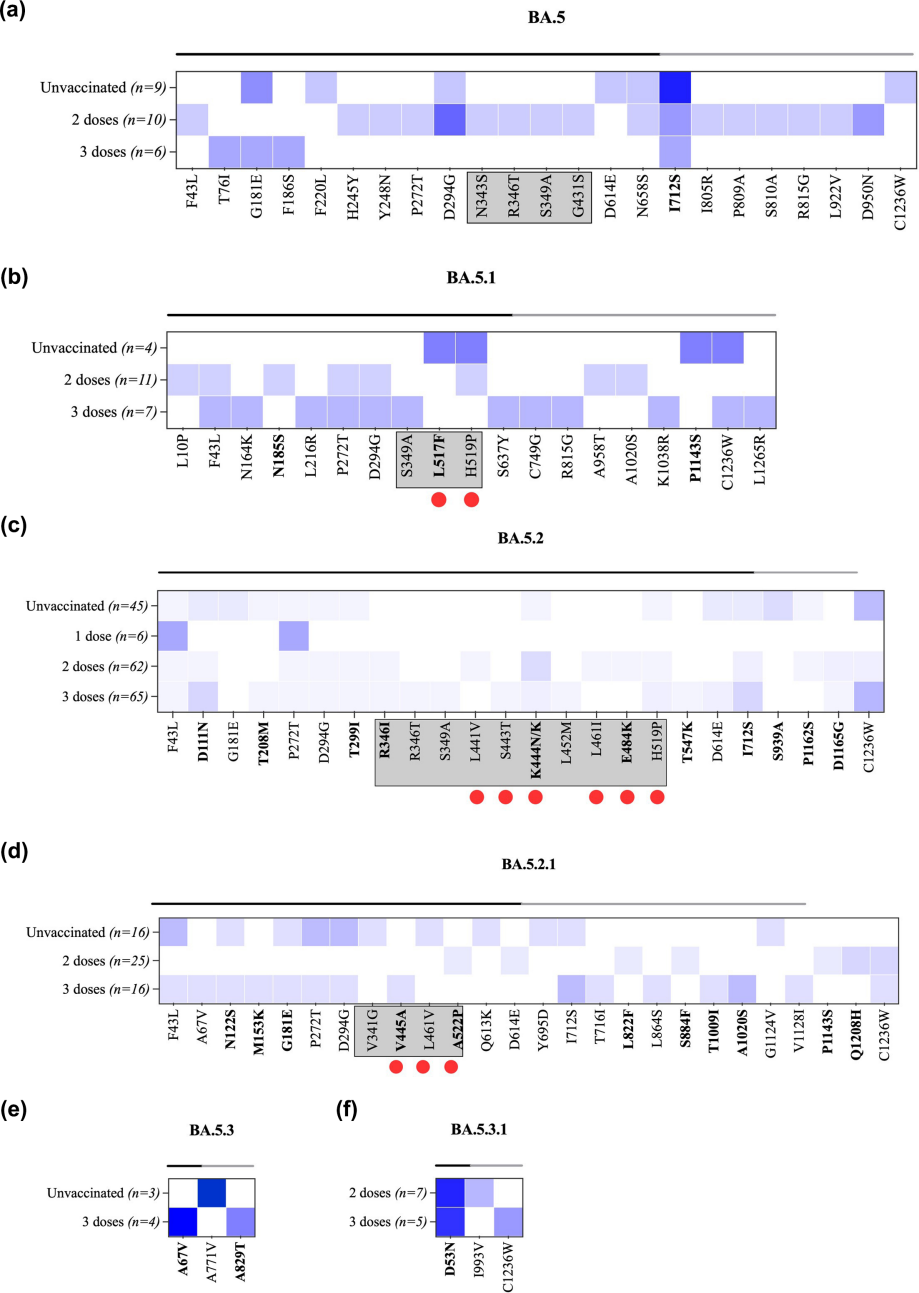
Heat maps demonstrating the prevalence of within-host mutations located in the S gene in (**a**) BA.5 sequences, (**b**) BA.5.1 sequences, (**c**) BA.5.2 sequences, (**d**) BA.5.2.1 sequences, (**e**) BA.5.3 sequences and (**f**) BA.5.3.1 sequences. The heat maps compare the prevalence of each mutation between unvaccinated and vaccinated individuals. Vaccinated groups were further subdivided based on the number of vaccine doses that they received into one-dose-vaccinated, two-dose-vaccinated and three-dose-vaccinated individuals. The X-axis represents the amino acid mutations in the S gene. The amino acid changes that lie in the S1 and S2 subunits are marked by black and grey lines on the top of the heat map, respectively. Mutations located in the RBD are boxed. Mutations identified at consensus levels are labelled in bold. The red circle denotes amino acid mutations in antigenic sites. The blue circle denotes mutations detected at global prevalence.

In BA.5 sequences, a total number of 22 sub-consensus mutations were detected. Four of these mutations were located in the RBD and had a prevalence of 10%: N343S, R346T, S349A and G431S (Fig. 7a). The most prevalent mutation found in BA.5 sequences is I712S in the S2 subunit (44% in unvaccinated, 20% in two-dose-vaccinated and 17% in three-dose-vaccinated individuals). Interestingly, this consensus-level mutation was not found in the GISAID sequences. In the BA.5.1 sub-lineage, 19 non-lineage mutations were observed in the S gene of both unvaccinated and vaccinated groups (Fig. 7b). Two of these mutations, L517F and H519P, are located in antigenic sites within the RBD region. H519P was found in both unvaccinated (25%) and two-dose-vaccinated (9%) individuals, whereas L517F was found only amongst unvaccinated individuals (25%). Another mutation, C1236W, was also found in unvaccinated (25%) and three-dose-vaccinated individuals (14%). Three mutations identified in BA.5.1 sequences, F43L, P272T and D294G, were found exclusively in two-dose- and three-dose-vaccinated individuals with a slightly higher prevalence amongst three-dose vaccinees (14%). Sequences belonging to the BA.5.2 sub-lineage have exhibited 25 non-lineage mutations. Seven of these mutations were found in antigenic sites in RBD: four mutations in two-dose vaccinees, one mutation in three-dose vaccinees and the remaining two that were found in both unvaccinated and vaccinated individuals. However, all these mutations were detected at very low prevalence in all groups (Fig. 7c). Sequences that belong to the BA.5.2 sub-lineage, BA.5.2.1, carried 15 low-prevalent sub-consensus mutations, three of which, V445A, L461V and A522P, are located in the S antigenic site (Fig. 7d). BA.5.3 and BA.5.3.1 sequences exhibited three mutations each, none of them located at antigenic sites (Fig. 7e, f). Overall, sub-consensus mutations were found in both unvaccinated and vaccinated individuals regardless of Omicron lineage. However, some of these mutations were found at higher prevalence amongst individuals who received three doses of vaccine. Moreover, 11% of these mutations were located in antigenic sites, indicating a possible impact of vaccine-induced immunity on triggering their emergence. Interestingly, though, the majority of these mutations were detected at low prevalence, which could also indicate a limited advantage.

## Discussion

Since late 2019, the novel coronavirus (SARS-CoV-2) has evolved rapidly and accumulated multiple mutations that led to the emergence of many VOC. The source of these variants is not fully elucidated; however, the uncontrolled virus replication in immunocompromised patients is believed to be a primary source [19,31, 32]. The replication of the virus in immunocompetent patients may also result in a dynamic within-host mutation pattern that changes over the course of infection [14]. The accumulation of within-host mutations in individuals with pre-existing immunity can contribute to the emergence of immune-escape mutations and subsequent newer VOC [33,34]. The latest SARS-CoV-2 variant, the Omicron variant, emerged in November 2019 and has since become the most prevalent variant worldwide [35]. In addition to its high contagiousness, the Omicron variant raised alarming concerns because of its distinct ability to evade the immune response, both in vaccinated subjects and previously infected individuals [5,36,42]. Furthermore, the BA.4 and BA.5 variants can evade the immune response resulting from previous infections with Omicron BA.1 and BA.2, making them susceptible to recurrent infections [43]. Therefore, the Omicron wave is distinctly characterized by its higher reinfection rate compared to previous waves, indicating unique evolution and immune escape mechanisms [12]. Given the role of within-host mutations in RNA viruses’ evolution, studying the within-host diversity of Omicron variants may help to reveal factors affecting its evolution and immune escape mechanism. In our study, we explored the within-host diversity in vaccinated individuals infected with different Omicron variants to determine the impact of vaccine-induced immunity on within-host diversity. We have also investigated the impact of vaccine-induced immunity on the emergence of within-host antigenic mutations.

The ability of Omicron variants to evade the immune response could be related to the heterogeneity of the within-host virus population. Therefore, we investigated the quasispecies diversity of the three main Omicron variants (BA.2, BA.4 and BA.5). Overall, Omicron variants exhibited a limited number of within-host mutations (*n*=70 mutations) regardless of the lineage, vaccination status, vaccine type or number of vaccine doses. This is not surprising considering that acute COVID-19 infections are known to harbour a limited number of within-host mutations [44]. To minimize the effect of patient age and disease severity on within-host diversity, samples were selected from non-hospitalized patients aged between 12 and 55 years. This may further explain the limited number of within-host mutations reported in our samples. Moreover, Omicron infections are characterized by shorter incubation periods and milder symptoms compared to previous VOCs. The relatively short incubation period of Omicrons (3.42 days) compared to Alpha (5.00 days), Beta (4.50 days) and Delta (4.41 days) variants may have restricted virus replication and the resultant number of within-host mutations [45]. The milder symptoms associated with Omicron infections are also expected to result in a lower number of within-host mutations, based on previous reports of milder COVID-19 infections having lower associated within-host diversity compared to severe infections [17,20, 46,48]. So far, there are a limited number of studies comparing within-host diversity between different SARS-CoV-2 lineages. Overall, available studies reported either minimal or no difference in within-host diversity between different VOCs (Alpha, Beta, Delta and Omicron). Interestingly, however, the diversity levels in VOC samples were found to be significantly higher compared to non-VOC samples [18]. To the best of our knowledge, none of the published studies have compared the within-host diversity between different Omicron lineages. One study, however, reported comparable within-host diversity levels between early Omicron lineages, BA.1 and BA.2 [18].

Vaccination is another factor that may affect the within-host virus diversity and evolution. In the second part of our analysis, we investigated the impact of vaccine-induced immunity on within-host diversity. Overall, we found no significant difference between unvaccinated and vaccinated individuals regardless of vaccine type or number of doses. Similar findings were reported in studies that compared the effect of mRNA vaccines (BNT162b2 and mRNA-1273) and the inactivated virus vaccine (CoronaVac) on within-host diversity [18,49]. Vaccination status had no impact on the number of non-synonymous mutations or on the neutral purifying selection of mutations observed in unvaccinated samples. Moreover, Gu *et al*. [49] reported a minimal impact of vaccine-induced antibody and T cell responses on within-host SARS-CoV-2 sequence diversification [49]. These findings suggest that vaccination does not increase exploration of SARS-CoV-2 protein sequence space and may not facilitate the emergence of viral variants. Exceptions to this observation were found amongst the BA.5 and BA.5.2.1 sub-lineages, which showed a significantly higher within-host diversity amongst the vaccinated samples in comparison to the unvaccinated, and the BA.2.75.2 sub-lineage which shows a higher diversity amongst the unvaccinated samples. However, further characterization based on vaccine type revealed minimal difference between the study groups. Collectively, these results suggest no significant impact of Omicron lineage nor vaccination status on the level of within-host diversity. Therefore, we investigated the impact of vaccine-induced immunity on the occurrence of within-host mutations in the antigenic sites that might confer the virus immune evasion advantage.

The world immunity against SARS-CoV-2 has grown drastically during the last 2 years. The latest data show that more than 70% of the global population were vaccinated, and more than 85% were infected [50]. The circulation of Omicron variants in considerably highly immunized populations is expected to trigger the emergence of immune-escape mutations to avoid naturally acquired or vaccine-induced immunity [51]. Therefore, we investigated the occurrence and the prevalence of within-host mutations located in the antigenic sites of the S protein.

A higher prevalence of non-synonymous within-host substitutions in the S gene of Omicron lineages and sub-lineages was observed in vaccinated individuals. This indicates the enhancement of the S-gene mutations amongst the vaccinated group, as other studies have suggested [38,39]. Additionally, the accumulation of non-lineage mutations with the prevalence of more than 50% amongst Omicron lineages and sub-lineages alarms us that the virus has mutated effectively enough that the second- and third-dose vaccinations were ineffective. Therefore, the build-up of these mutations could be a reason for upcoming lineages or variants. Specifically, nine antigenic mutations were detected amongst the sequences, which may directly play a crucial role in the increased transmissibility and immune escape property of the virus. Since these mutations were found in vaccinated groups in prevalence, neutralizing those mutations would give knowledge on the immune-escape mutations and vaccine efficacy. However, it is effectively impossible to precisely describe the full influence of the Omicron variants’ S protein mutations on the present vaccines in the world’s populations. This is due to several reasons, including different immune responses elicited in the same individual by vaccination, regardless of belonging to the same or different vaccine types. Individuals vaccinated by the same vaccine type may be further characterized by race, sex, age and medical conditions, all of which led to the production of different sets of antibodies (different epitopes). Moreover, the inability to entirely control various experimental conditions limits the reliability of the statistical examination over the populations. To date, vaccination has been proven to be the most effective approach for COVID-19 prevention and control. Nevertheless, the 70 amino acid mutations in the S protein suggest that the emergence and evolution of Omicron may be induced by vaccination. Thus, the rapid development of within-host mutations will improve the virus’s ability to escape the presently administered vaccines.

Non-synonymous S mutations R346T, F486S and D1199N in BA.2 and V3G and N658S in BA.5 were detected in our data with prevalence consistent with the global consensus. Only two recognized mutations of concern, L452R and K417T, were found in our sequences. L452R was found in our BA.2 sequences with notable prevalence. This is interesting since consensus shows no global prevalence for this mutation amongst BA.2 sequences but is recorded in high levels in BA.4 and BA.5 sequences. This may be indicative of the accumulation of this mutation leading up to the emergence of BA.4 and BA.5 variants. None of the RBD antigenic mutations that were observed in our findings, with the exception of H519P, were fixed in the infected human population, suggesting a purifying selection pressure. These mutations may be accumulated over time and result in newly emergent variants. Similar observations were made when deep sequencing of 90 nasopharyngeal samples showed that many mutations associated with the establishment of SARS-CoV-2 globally were present at varying frequencies in a majority of the samples collected at the time of the virus’ initial detection in the USA. A subset of mutations that emerged months later in consensus sequences were detected as sub-consensus members of intra-host populations. Previous studies have reported this [34]. Their location of the sub-consensus mutations within antigenic sites could result in subsequently compromised immune protection in the host. The effect of these mutations on the virus-neutralizing activity of the host immune system can provide a better insight into the significance of their detection.

In conclusion, within-host diversity in Omicron samples does not seem to be affected by Omicron lineage or vaccination status. Vaccinated individuals, however, carried non-synonymous mutations that may reduce virus neutralization by vaccine-induced immunity. Further analysis of the effect of these mutations on virus neutralization effectiveness should be performed. Moreover, detected mutations with low or no global consensus should be monitored as they may be anticipated mutations of concern.

## Supplementary material

10.1099/jgv.0.002108 Uncited Supplementary Material 1.

10.1099/jgv.0.002108 Uncited Table S1.

## Abbreviations

MAFs: minor allele frequencies
ONT: Oxford Nanopore Technology
RBD: receptor-binding domain
S: spike
VOC: variants of concern
WHO: World Health Organization
