# Supplementary material for: The impact of pre-existing immunity on the emergence of within-host immune-escape mutations in Omicron lineages
Source: J Gen Virol. 2025 May 13;106(5):002108. doi: 10.1099/jgv.0.002108 (PMC12075854; doi:10.1099/jgv.0.002108)
Supplement: Uncited Supplementary Material 1. [file jgv-106-02108-s001.pdf]

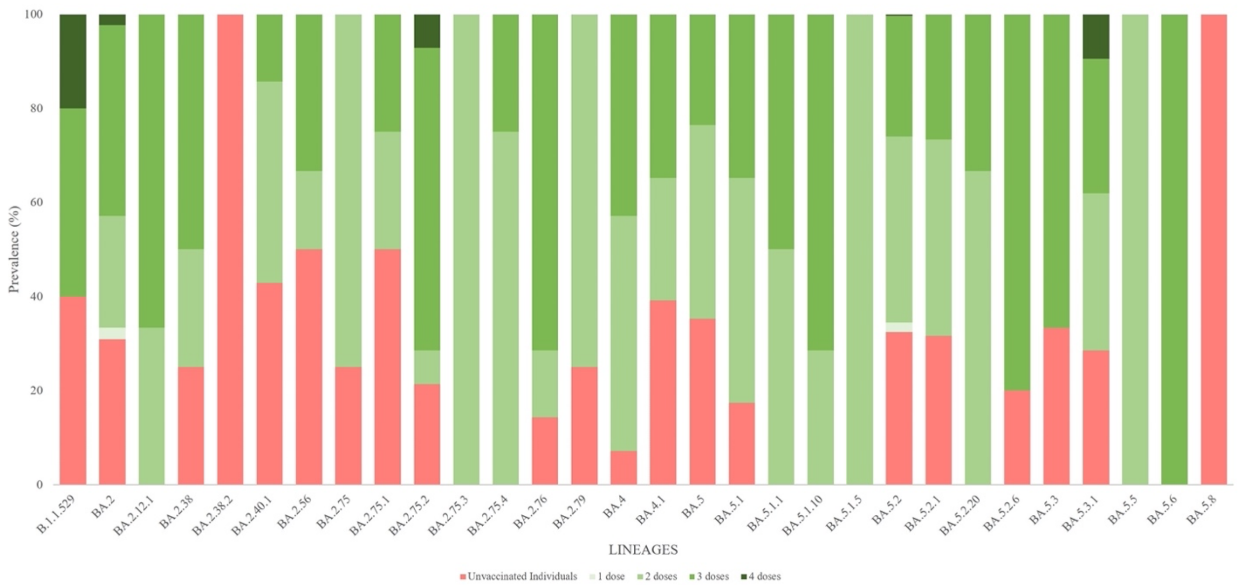

Supplementary figure 1: Prevalence of omicron lineages among vaccinated and unvaccinated individuals. The graph demonstrates the prevalence of the most prevalent omicron lineages in unvaccinated individuals as well as individuals vaccinated with one-dose, two-doses, three-doses, and four-doses. The colors represent the vaccination status of individuals: green for vaccinated and red for unvaccinated individuals.

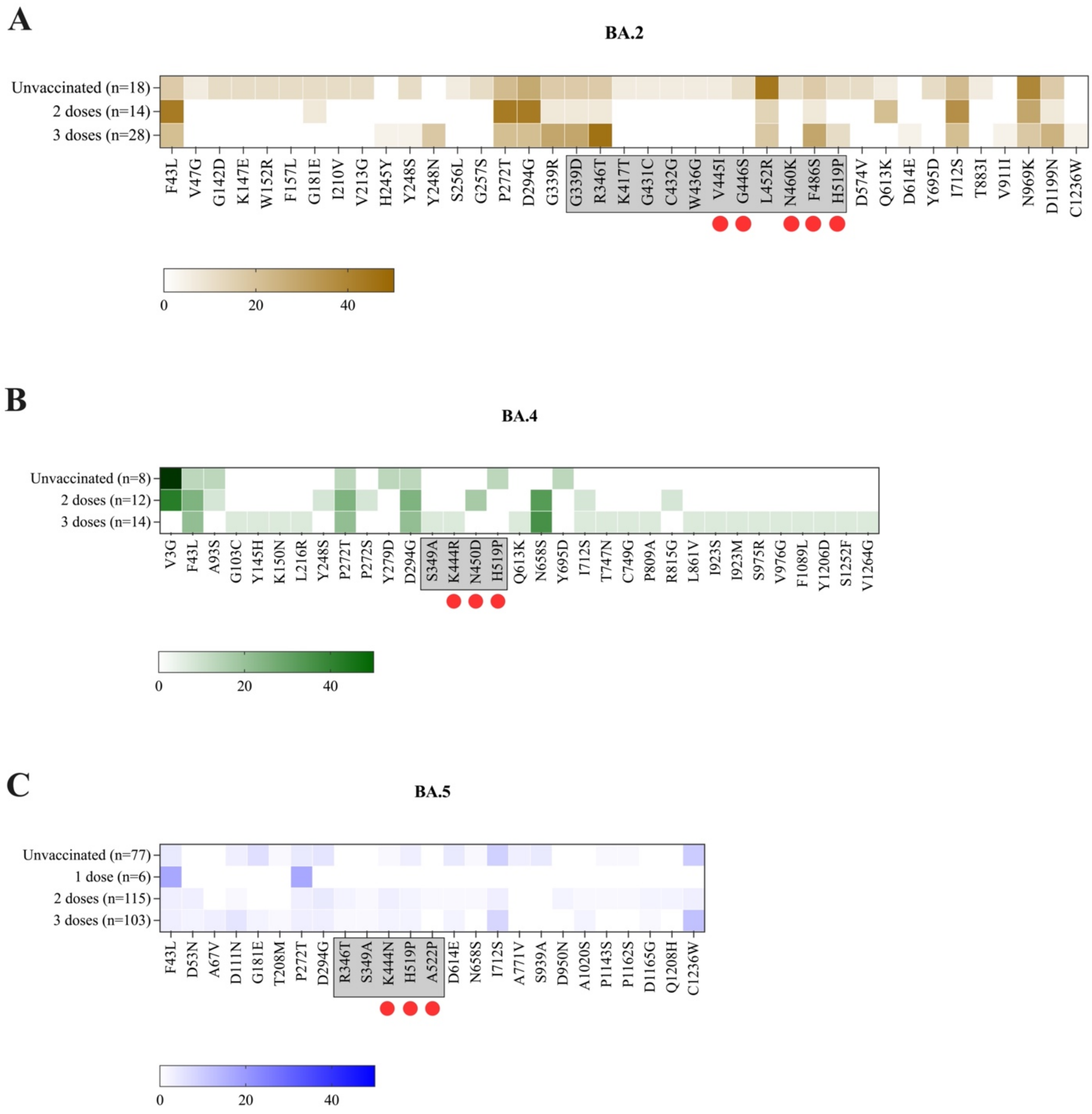

Supplementary figure 2: Heat maps demonstrating the prevalence of within host mutations located in Spike (S) gene in **(A)** BA.2 lineage and sub-lineage sequences; **(B)** BA.4 lineage and sub-lineage sequences; **(C)** BA.5 lineage and sub-lineage sequences. The heat maps compare the prevalence of each mutation between unvaccinated and vaccinated individuals. Vaccinated groups were further subdivided based on number of vaccine doses they received into one-dose vaccinated, two-dose vaccinated, and three-dose vaccinated individuals. The X- axis represent the amino acid mutations in S-gene. Mutations located in the receptor binding domain (RBD) are boxed. Mutations identified at consensus levels are labeled in bold. The red circle denotes amino acid mutations in antigenic sites.
